# From Repair to Replacement: Ring Detachment and Secondary Annular Injury During Transcatheter Mitral Valve-in-Ring Procedure

**DOI:** 10.1016/j.jaccas.2025.103404

**Published:** 2025-04-02

**Authors:** Andrés Felipe Ochoa-Díaz, María Carolina Manzur-Barbur, Luis Enrique Giraldo, Nicolas Ariza-Ordoñez, Yilmar Meza-González, Darío Echeverri, Jaime Cabrales

**Affiliations:** a School of Medicine and Health Sciences, Universidad del Rosario, Bogota, Colombia; b Interventional Cardiology Department, LaCardio, Bogota, Colombia

**Keywords:** cardiac surgical procedures, mitral valve annuloplasty, mitral valve stenosis

## Abstract

**Objective:**

The present case portrays an infrequent complication of transcatheter mitral valve replacement (TMVR) in a patient with a previous surgical mitral valve repair with ring annuloplasty and an Alfieri stitch, who developed mitral stenosis and heart failure.

**Key Steps:**

After transseptal puncture, balloon atrial septostomy and mitral balloon valvuloplasty were performed. The transcatheter valve was positioned and deployed through a partial dehiscence on the anterior aspect of the prosthetic ring, probably caused by the balloon valvuloplasty or undetected in the preprocedural imaging assessment. This led to prosthetic ring detachment and dislocation, native annular tearing, migration of the transcatheter prosthesis, and subsequent hemodynamic collapse requiring open surgery.

**Potential Pitfalls:**

In TMVR, prosthesis misalignment or malposition may cause annular stress during deployment and tearing of the native annulus, prosthesis embolization from suboptimal anchoring, and cardiac tamponade from left ventricular rupture, often requiring emergency surgical intervention.

## Graphical Abstract

**Figure:**
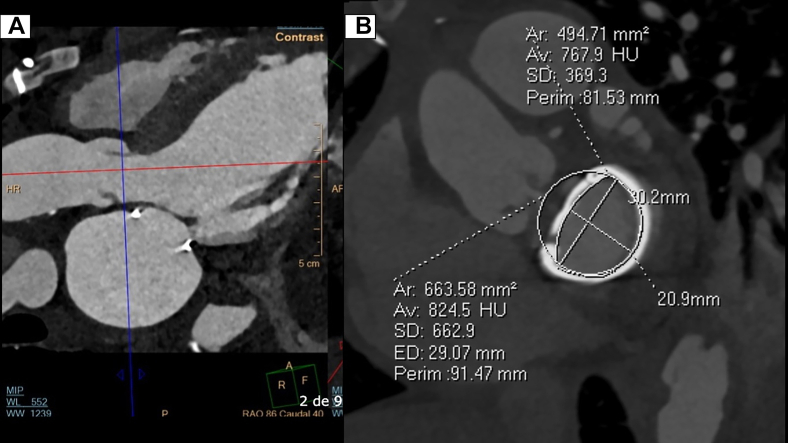

Surgical mitral valve repair is preferred over replacement because of better long-term outcomes and the ability to postpone the need for valve replacement, particularly in younger patients.^1^ Surgical mitral valve repair can be performed using multiple techniques, including annuloplasty, which is a procedure designed to reshape and stabilize the mitral annulus by attaching an annuloplasty ring to its atrial aspect.^2^ The objective of surgical mitral valve repair is to avoid reoperation for mitral valve replacement, which imposes a higher mortality risk when compared with the first procedure.^3^ In patients with mitral valve dysfunction after surgical mitral valve repair with ring annuloplasty, and at high surgical risk, transcatheter mitral valve replacement (TMVR), valve-in-ring, offers a viable and less invasive alternative.^2^

**Take-Home Messages**

• Annular injury is a rare complication of transcatheter mitral valve-in-valve procedures, and it contributes to malposition and dysfunction of the prosthesis.
• Annular injury increases the risk of cardiac tamponade by commissure tear, highlighting the need for emergency surgery repair.

The first valve-in-ring TMVR was performed in 2011.^4^ By 2020, more than 3,597 TMVR procedures had been performed in the United States, and most of these procedures used balloon-expandable devices.^5^ Annular injury caused by inadequate prosthesis deployment is a rare complication that can lead to valvular malposition, which requires open heart surgery.^6^^,^^7^

## Case Summary

A 57-year-old woman was referred to our center (LaCardio, Bogotá, Colombia) with a 2-month history of shortness of breath, bilateral lower limb edema, and several arterial ulcers in the lower limbs with diminished perfusion. Her medical history included the following: ischemic cardiomyopathy, with a left ventricular ejection fraction (LVEF) of 26%; and significant mitral regurgitation, with a mildly dilated mitral annulus with a diameter of 38 mm, restriction of the posterior leaflet, an effective regurgitant orifice area of 0.32 cm^2^ by the proximal isovelocity surface area method, and a regurgitant volume of 45 mL. Six months before the index consultation, she underwent coronary artery bypass grafting (CABG) (left internal mammary artery [LIMA] to left anterior descending [LAD] artery and saphenous venous graft [SVG] to second obtuse marginal [OM]) and surgical mitral valve repair with annuloplasty (using a complete semirigid prosthetic ring [CG Future #32, Medtronic]) and an edge-to-edge technique (also known as the Alfieri stitch). Surgical findings included retraction of P2 and prolapse of A2 scallops.

On admission, she received a diagnosis of acute decompensated heart failure, with a wet-warm profile. Guideline-directed medical therapy and intravenous diuretic management were promptly initiated. A transesophageal echocardiogram revealed moderate left ventricular dilation with global hypokinesis and an LVEF of 15% to 20%. The mitral valvuloplasty was found to be dysfunctional, exhibiting restricted opening with a mean transvalvular gradient of 15 mm Hg, a peak velocity of 2.2 m/s, and a mitral valve area of 1 cm^2^ (calculated by the pressure half-time method), with no significant regurgitation or paravalvular leaks suggestive of ring dehiscence (Videos 1A to 1E). Systolic pulmonary artery pressure was estimated at 62 mm Hg, which, along with the other findings, indicated hemodynamically significant mitral stenosis.

**Figure 1:**
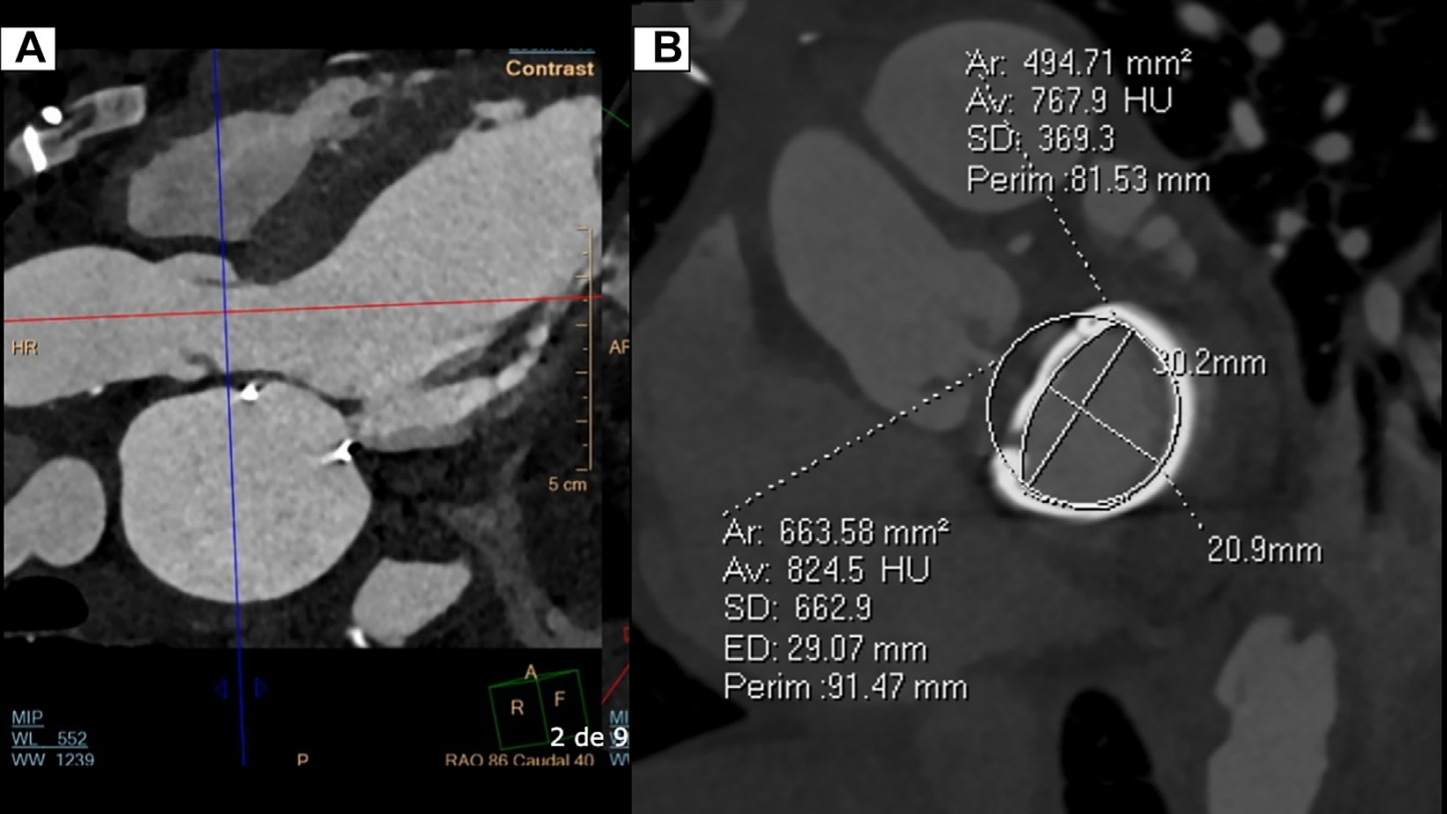
Preprocedural Contrast-Enhanced Cardiac Computed Tomography (A) A 3-chamber view showing the left ventricular outflow tract and mitral valvuloplasty ring without any evidence of dehiscence. (B) Simulation of a transcatheter mitral valve implantation with a neo-left ventricular outflow tract of 1.7 cm^2^. AP = anteroposterior; Ar = area; Av = average; ED = estimated diameter; F = feet; HU = Hounsfield unit; MIP = maximum intensity projection; P = posterior; Perim = perimeter; R = right; RAO = right anterior oblique; SD = standard deviation; WL = window level; WW = window width.

Coronary angiography showed an occluded aortocoronary SVG to the second OM and a patent LIMA graft to the LAD artery, with native multivessel coronary disease, including total proximal occlusion of the right coronary artery with collateral filling, middle segment occlusion of the LAD artery, and distal occlusion of the second OM artery (Videos 2A, 2B, 3A, 3B, and 4). A preprocedural evaluation included a cardiac computed tomography scan with a simulated neo-left ventricular outflow tract area of 1.7 cm^2^ (Figures 1A and 1B). The heart team decided to proceed with percutaneous coronary intervention for the native vessels, followed by TMVR (valve-in-ring), by taking into account the prohibitive surgical risk for mitral valve replacement and CABG redo (European System for Cardiac Operation Risk Evaluation II [EuroSCORE II], 38.6% for operative mortality, driven by extracardiac arteriopathy, previous cardiac surgery, LVEF <20%, and pulmonary hypertension).

## Procedural Steps

### Transseptal puncture, balloon atrial septostomy, and mitral balloon valvuloplasty

Using a right femoral venous sheath introducer and a 0.035-inch J-tipped guidewire, the valve sheath introducer and the steerable Agilis catheter (Abbott) were inserted. The transseptal needle was advanced into the right atrium, and the puncture site was identified under fluoroscopic and transesophageal guidance. After successfully performing the puncture (Videos 5A to 5C), the Agilis catheter (Abbott) was advanced into the left atrium and oriented in front of the mitral orifice. Subsequently, a J-preshaped stiff wire Confida (Medtronic) was advanced to the apex. A 14 × 40 mm Atlas balloon (BD)was then introduced for the atrial septostomy and was advanced into the left ventricle for performance of mitral balloon valvuloplasty (Videos 6A and 6B).

### Placement and deployment of the transcatheter valve prosthesis in the mitral position

Once the mitral transcatheter valve (Sapiens 29-mm valve, Edwards Lifesciences) was properly positioned within the deployment system, it was advanced to the annular location (Video 7). The high support guidewire was repositioned after the balloon valvuloplasty. The prosthesis was advanced and deployed through a partial dehiscence of the anterior aspect of the prosthetic ring, which may have been caused by the balloon valvuloplasty or could have preexisted and gone undetected in the preprocedural imaging. This malposition led to prosthetic ring detachment and dislocation, native annular tearing, and prosthetic valve malposition (Videos 8A and 8B). The complication led to torrential mitral regurgitation with migration of the prosthesis to the left atrium, with hemodynamic collapse.

### Management of malposition of the mitral valve-in-ring prosthesis and corrective approach

Given the patient’s hemodynamic instability and our inability to retrieve the device and repair the suspected annular injury percutaneously, an open surgical approach was considered. A median sternotomy was performed, followed by complex dissection to release pleuropericardial adhesions. A left atriotomy was conducted. On direct surgical inspection, there was no annular rupture or hemopericardium. It was confirmed that the primary lesion was an annular tear of the anterolateral commissure, with 90% dehiscence of the prosthetic ring. During the procedure, a free-floating prosthesis in the left atrium over the high-support guidewire was removed (Video 9).

Reinforcement of the tear in the anterolateral commissure was performed with U-shaped polypropylene (Prolene, Ethicon) sutures over pledgets, followed by resection of the anterior and posterior leaflets and the dysfunctional subvalvular apparatus. Ring measurement was performed, followed by fixation of an Epic Plus biological prosthesis (Abbott) in the mitral position (27 mm) with separated polyester (Ethibond, Ethicon) 2-0 sutures, and reinforcement at the anterolateral commissure with Ethibond (Ethicon) 2-0 sutures over pledgets. The procedure ended with closure of the post-atrioseptostomy atrial septal defect. At 48 hours after the surgical procedure, the patient died of severe left ventricular dysfunction and mixed vasoplegic and cardiogenic shock.

## Potential Pitfalls

### TMVR misalignment

The TMVR device can be positioned at an angle not perpendicular to the annuloplasty ring. This malposition can lead to excessive stress on both prosthetic and native rings, with resulting detachment and tearing. Achieving coaxial alignment during TMVR is crucial for ensuring precise positioning of the prosthesis within the mitral annulus. Attaining this alignment is considered challenging in procedures performed through the transseptal approach.^8^

### Prosthesis embolization

Deployment of the prosthesis in a suboptimal position can reduce the friction between the transcatheter prosthetic mitral valve and the surrounding tissue that is essential for proper anchoring. This situation increases the risk of migration, particularly given the significant pressure differential between the left ventricle and the left atrium during systole. This risk can be mitigated by ensuring conical deployment (20%-30% toward the left atrium and 70%-80% toward the left ventricle), which avoids parallel positioning that favors atrial migration. In cases of embolization, open surgical replacement is the recommended treatment.^9^

### Cardiac tamponade secondary to left ventricular rupture

Rupture of the mitral anterolateral commissure can affect nearby structures in the left atrioventricular groove, such as the coronary sinus and the left circumflex artery. Rupture of these structures can lead to cardiac tamponade, significantly increasing the risk of mortality and indicating the need for emergency surgery.^10^

## Conclusions

Annular injury during transcatheter mitral valve-in-ring procedures is a rare complication with an undefined incidence. It can lead to suboptimal deployment and prosthetic dysfunction, rendering the endovascular approach ineffective and increasing the risk of left ventricular rupture or prosthesis embolization. In such cases, open surgery becomes necessary, shifting the focus to valve replacement.

## Funding Support and Author Disclosures

The authors have reported that they have no relationships relevant to the contents of this paper to disclose.

## Appendix

Video 1 Preprocedural Echocardiography Assessment (A) Parasternal-long axis, (B) apical 4-chamber, and (C) midesophageal bicommissural views documenting, in color Doppler imaging, diastolic acceleration of transmitral flow compatible with stenosis, along with a trivial regurgitation jet. (D) Continuous-wave Doppler imaging at the mitral prosthesis with a 15 mm Hg transvalvular mean gradient and a pressure half-time of 220 ms. (E) Continuous-wave Doppler imaging at the mitral prosthesis with a trivial regurgitation jet.

Video 2 Coronary Angiography (A) The left anterior projection shows proximal total occlusion of the right coronary artery. (B) The cranial left anterior projection indicates a severe lesion of the proximal segment of the left anterior descending and competitive flow with the internal mammary artery graft.

Video 3 Graft Angiography (A) The right internal mammary artery is shown without anastomosis. (B) The left internal mammary artery is shown anastomosed to the left anterior descending artery.

Video 4 Fluoroscopic View Imaging shows the semirigid CG Future mitral annuloplasty ring (Medtronic), the prosthetic ring used in the first mitral valve repair.

Video 5 Valve-in-Ring Procedure: Transseptal Puncture and Balloon Septoplasty (A) Transseptal puncture is observed. (B) A high-support guidewire (Confida, Medtronic) is advanced into the ventricular cavity and crosses the mitral plane. (C) Septoplasty is performed with a balloon.

Video 6 Valve-in-Ring Procedure: Mitral Balloon Valvuloplasty (A) The system positioned in the inferior vena cava for deployment of the balloon-expandable valve. There are 2 high-support guidewires: the first holds the predilation balloon, and the second holds the valve to be implanted(B) Successful mitral balloon valvuloplasty is observed.

Video 7 Passage Through the Interatrial Septum of the Valve Release System and Its Positioning on the Mitral Annular Plane

Video 8 Annular Injury (A) Deployment of the balloon-expandable valve leading to detachment of the prosthetic ring in an anterior-to-posterior direction. (B) Prosthetic ring trapped between the transcatheter heart valve and the native mitral annulus.

Video 9 Surgical and Fluoroscopic Views (Left) Surgical view The dislocated and torn CG Future mitral annuloplasty ring (Medtronic) is visible, along with the balloon-expandable valve. (Right) Fluoroscopic view of both mentioned structures.
